# Successful ablation of premature ventricular complexes arising from the posteromedial papillary muscle using Pulse Field Ablation

**DOI:** 10.1016/j.hrcr.2025.01.013

**Published:** 2025-01-30

**Authors:** Nestor Vasquez, Jonathan Chrispin

**Affiliations:** Department of Medicine, Division of Cardiology, Johns Hopkins University, Baltimore, Maryland

**Keywords:** Premature ventricular complexes, Pulse field ablation, Papillary muscle ablation, Posteromedial papillary muscle, Catheter ablation

**Key Teaching Points**

• The left ventricular papillary muscles (PMs) are intracavitary structures that can be the source of ventricular arrhythmias; however, complex anatomy, variable site of origin and exit site, inadequate catheter stability, and contact force can limit catheter ablation success at these sites.
• Pulse field ablation (PFA) can be a valuable ablation modality to successfully target arrhythmias arising from the PMs, especially when guided by intracardiac echocardiography and electroanatomic mapping.
• Larger studies are needed evaluating the efficacy and safety of PFA to treat arrhythmias originating from the PMs.

## Introduction

The left ventricular papillary muscles (PMs) are intracavitary structures that can be the source of ventricular arrhythmias in patients with and without structural heart disease.^1^ Catheter ablation can be very effective and safe for treating these arrhythmias; however, success rates have been reported to be lower compared with arrhythmias originating from the ventricular outflow tracts.^1^^,^^2^ Complex anatomy, variable site of origin and exit site, inadequate catheter stability, and contact force are among the reasons why ablating arrhythmias from the PMs can be challenging despite the use of contact force–sensing catheters and intracardiac echocardiography (ICE).^1^^,^^2^

Pulse field ablation (PFA) is a novel ablation modality that applies a series of punctuated high-voltage electrical energy fields, causing irreversible electroporation with subsequent destabilization of cell membranes, leading to cell death.^3^ Clinical trials have shown the efficacy of PFA for pulmonary vein isolation.^4^, ^5^, ^6^ There is growing interest in the use of PFA to treat ventricular arrhythmias, including intracavitary structures such as PMs. Perhaps PFA could account for some of the challenging limitations described while ablating ventricular arrhythmias originating from the papillary muscles. We present a case of premature ventricular complexes (PVCs) arising from the posteromedial PM successfully ablated using radiofrequency energy and PFA.

## Case report

**Figure 1:**
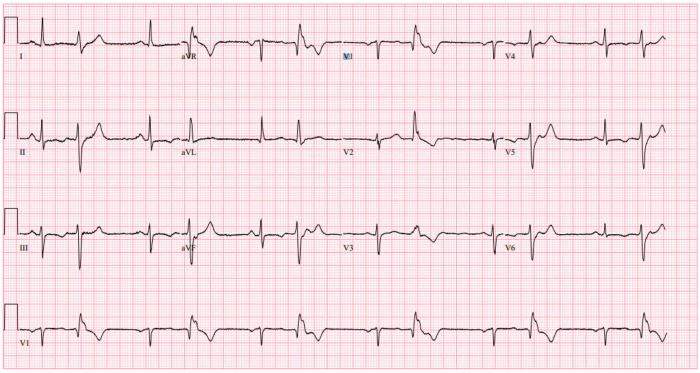
12-Lead electrocardiogram with premature ventricular complexes (PVCs) in bigeminy pattern.

A 66-year-old woman with a medical history of hyperlipidemia, migraines, and breast cancer was referred by her primary care provider for palpitations and bradycardia. A 12-lead electrocardiogram was obtained in the clinic, which showed sinus rhythm with frequent PVCs in a bigeminy pattern. The PVC morphology was right bundle branch block with a qR pattern in V1, transition in V4, superior axis, positive in lead I and aVL, QRS duration of 165 msec, maximal deflection index 0.44 (Figure 1).

A 7-day cardiac monitor was obtained with a PVC burden of 36%, which was associated with symptoms of palpitations and fatigue. Subsequent workup included an echocardiogram, which showed an ejection fraction (EF) of 40% with mild diffuse hypokinesis of the left ventricle, normal right ventricular size and function, and mild mitral regurgitation. A cardiac magnetic resonance imaging showed a left ventricular EF of 36%, normal chamber sizes, and no late gadolinium enhancement to suggest fibrosis.

Treatment with metoprolol succinate 25 mg daily was started, and a repeat 7-day cardiac monitor 6 weeks later showed a similar PVC burden of 36%, with a greater frequency of nonsustained ventricular tachycardia episodes. Thus, metoprolol was stopped, and verapamil 120 mg twice daily was started. A repeat 7-day cardiac monitor obtained a month later still showed a PVC burden of 27% associated with symptoms, so the patient was referred to our center for a PVC ablation.

**Figure 2:**
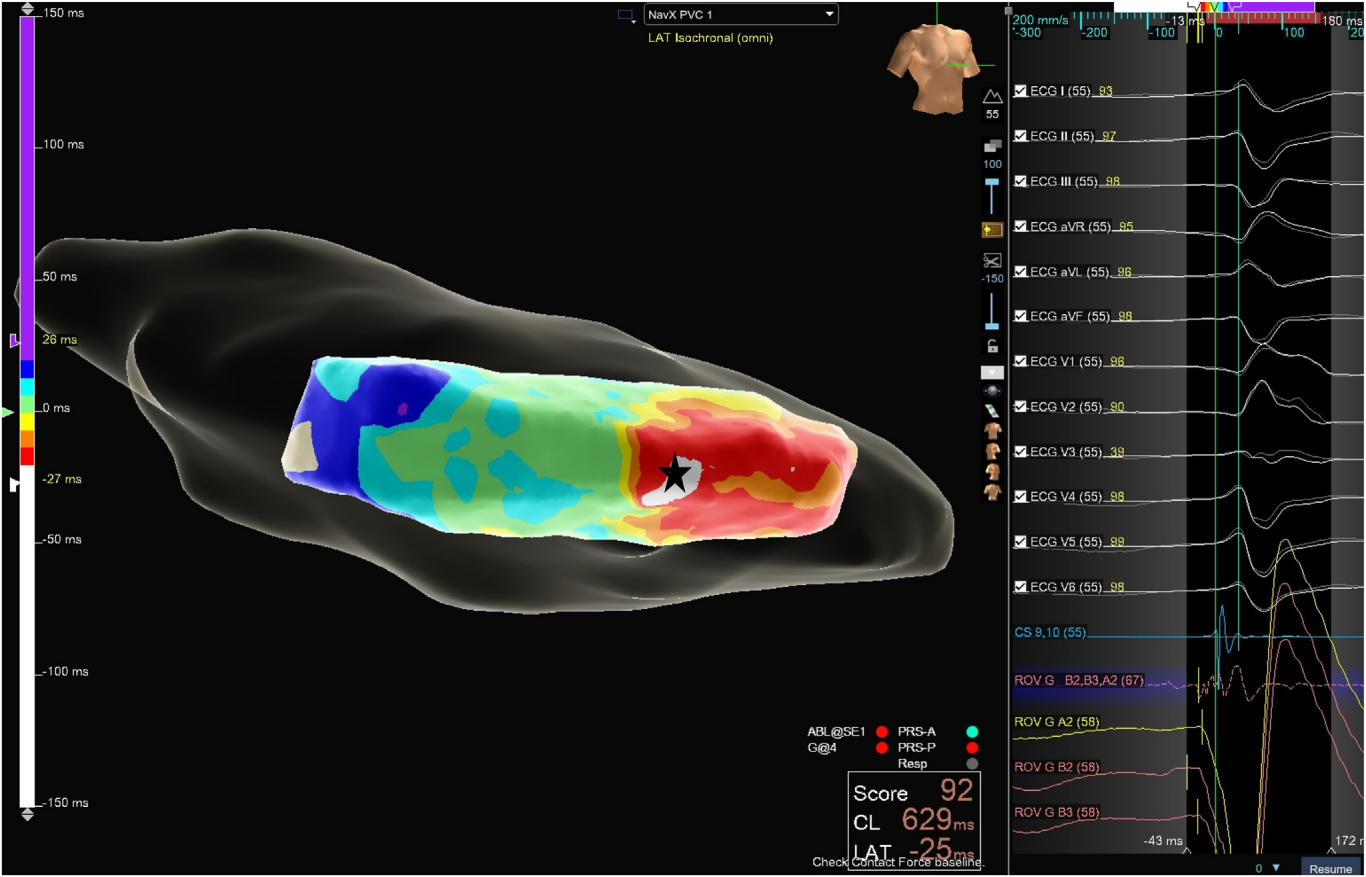
Premature ventricular complexes (PVC) activation map of the left ventricle showing earliest activation site at the posteromedial papillary muscles (PM) with earliest local electrogram (EGM) minus 25 milliseconds.

Following informed consent, the patient presented to the electrophysiology laboratory. Light sedation was induced and maintained by the anesthesiology team. After femoral venous vascular access, an electroanatomic map of the left ventricle was created by a trans-septal approach with the Advisor HD grid mapping catheter (Abbott, Abbott Park, IL) and the EnSite X EP System (Abbott, Abbott Park, IL). Given the readily frequent PVCs of the same morphology, activation mapping was used to bracket the earliest activation in the left ventricle. The activation mapping suggested the earliest activation at the septal aspect of the posteromedial PM. This area was bracketed using the Advisor HD grid mapping catheter, with the earliest local electrogram to QRS onset being minus 25 milliseconds and a QS pattern on unipolar electrogram mapping (Figure 2). Thus, the decision was made to deliver radiofrequency ablation lesions at the septal aspect of the posteromedial PM.

**Figure 3:**
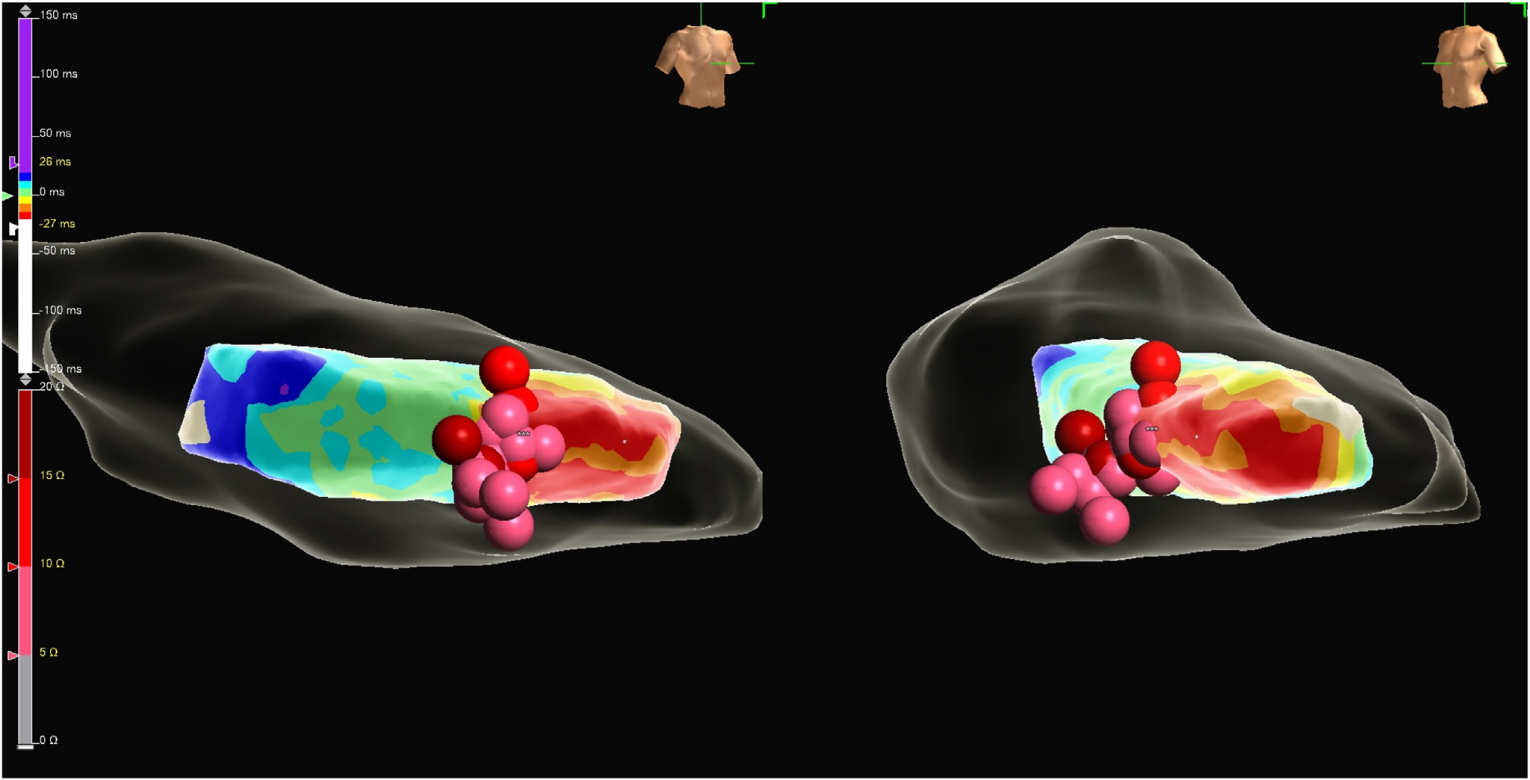
Radiofrequency ablation lesions delivered at the posteromedial papillary muscles (PM). Premature ventricular complexes (PVC) suppression was seen at the “∗∗∗” marked site.

The Advisor HD grid mapping catheter was exchanged for the sensor-enabled bidirectional TactiFlex Ablation irrigated catheter (Abbott, Abbott Park, IL). A total of 12 radiofrequency ablation lesions were delivered at this site using a power of 40–45 Watts, a duration of 45–90 seconds per lesion, and adequate contact force. ICE was also used to verify catheter stability and adequate contact. PVCs were effectively suppressed on the 10th radiofrequency ablation lesion (Figure 3). However, after 15 minutes into a waiting period, the PVCs recurred in a patten of bigeminy.

**Figure 4:**
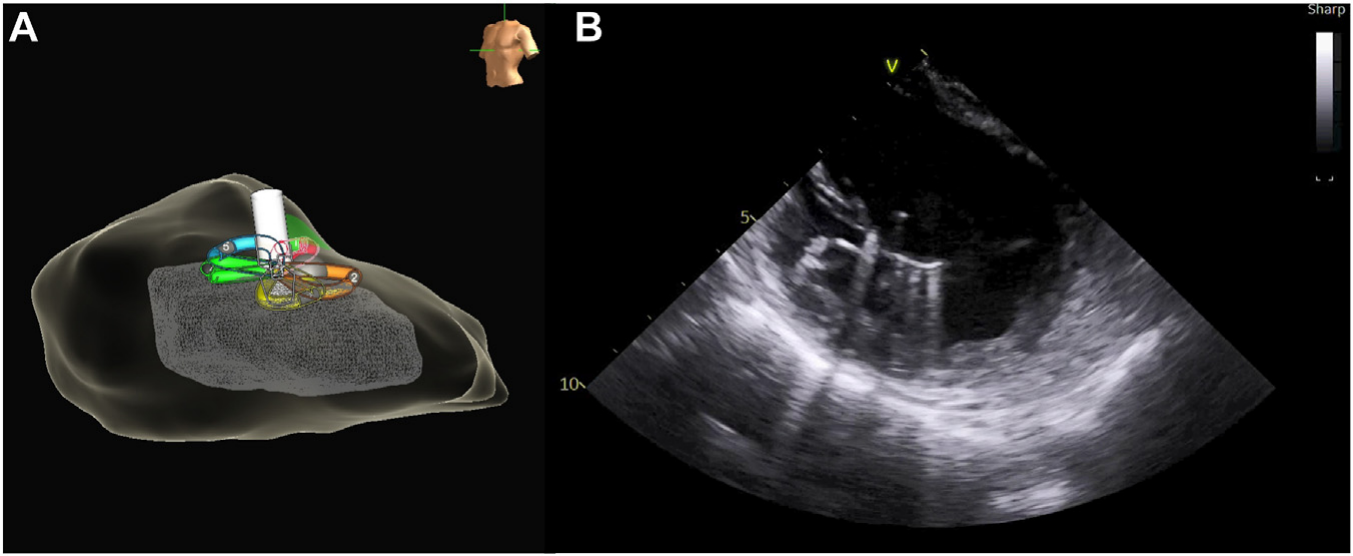
**A:** Farawave pulse field ablation (PFA) ablation catheter in flower configuration sitting on top of the posteromedial papillary muscles (PM). **B:** Intracardiac echocardiography (ICE) showing the Farawave PFA ablation catheter in flower configuration sitting on top of the posteromedial PM before ablation.

**Figure 5:**
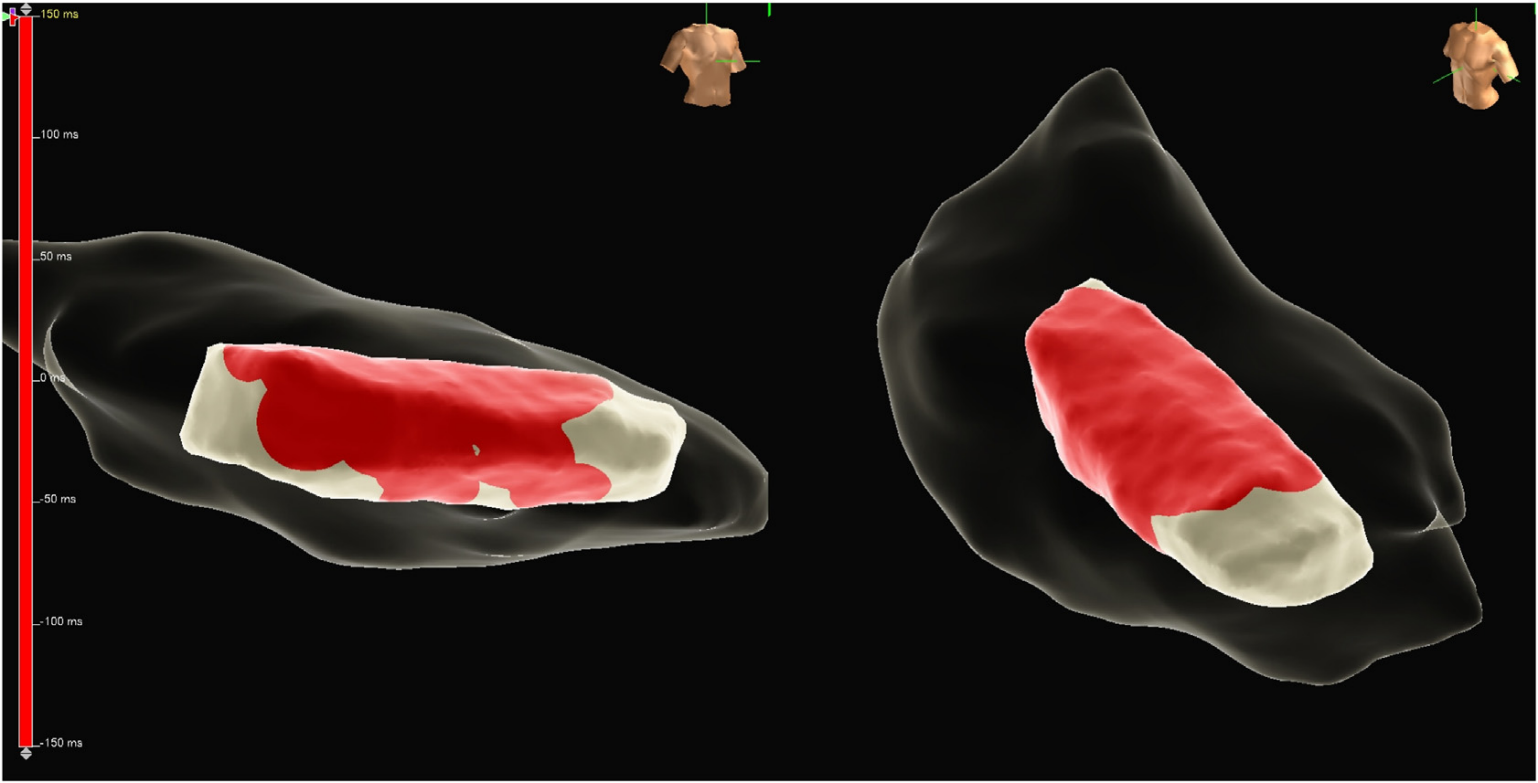
EnSite X EP System showing the Farapulse pulse field ablation (PFA) catheter ablation lesion projection in *red* on the posteromedial papillary muscles (PM).

Given the persistence of PVCs despite adequate radiofrequency ablation lesion delivery, the decision was made to switch to PFA for PVC ablation at this site. The Agilis sheath (Abbott, Abbott Park, IL) was exchanged over a wire for the Faradrive sheath (Farapulse PFA System, Boston Scientific, Natick, MA). A Farawave ablation catheter (Farapulse PFA System, Boston Scientific, Natick, MA) was advanced into the left ventricle. The Farawave ablation catheter was set to flower configuration and advanced to the septal aspect of the posteromedial PM using fluoroscopy, EnSite X EP System, and ICE guidance (Figure 4, Video 1). An initial PFA application was delivered at this site, noting immediate PVC suppression. A total of 10 PFA applications were delivered to ensure appropriate PVC ablation (Figure 5). No ventricular tachycardia or ventricular irritative firing was noted during application delivery. Following a 20-minute waiting period, no PVCs were noted. After sheath removal and hemostasis, the patient was transported to the recovery unit, where no PVCs were noted.

The patient was discharged off verapamil the same day of the procedure. A 3-day cardiac monitor was obtained 2 weeks later with a PVC burden of <1%. No runs of ventricular tachycardia were reported. Moreover, a repeat echocardiogram 6 weeks after the procedure showed improved left ventricular systolic function, with an EF of 45% to 50%, normal right ventricular systolic function, and trace mitral regurgitation. Since the procedure, the patient has had marked improvement in her symptoms without complications.

## Discussion

The posteromedial PM is a left ventricular intracavitary structure that originates from the inferoseptal wall and is connected to the mitral valve leaflets via chordae tendinae.^1^ Achieving catheter stability can be challenging because of the mobile nature of PMs.^2^ PM arrhythmias can also exhibit multiple QRS morphologies because of changing exit sites while ablating, which, along with PM thickness and potential deep intramural sites of origin, can further limit ablation success.^7^^,^^8^ Moreover, PM anatomic variations and a complex Purjinke fiber–muscular interface within the PM make ablation at this site particularly difficult.^2^ Prior studies have described lower ablation success rates for PM arrhythmias compared with other ablation sites.^7^^,^^9^^,^^10^ A recent study at an experienced center showed a better acute success rate, although with a long-term success rate of 82% before repeat ablation.^8^ Although success rates seemed to be independent of the use of contact force-sensing catheters, perhaps the use of other tools such as ICE contributed to the improvement in ablation outcomes.^8^^,^^11^ In addition, cryoablation has also emerged as an important alternative to address catheter stability and adequate catheter contact.^12^ However, other limitations, including lower lesion depth and adequate catheter tip visualization on electroanatomic mapping, are to be considered.^1^

PFA is a novel ablation modality that has shown efficacy for atrial fibrillation ablation and can potentially address some of the limitations encountered with radiofrequency or cryoablation at the PMs.^4^ A preclinical study in a porcine model using a lattice-tip PFA catheter to ablate the PMs showed, on average, 6-mm lesion depth using 3 to 5 repeated PFA applications at this site.^13^ Lesions with poor contact resulted in shallower lesions; otherwise, there were no differences in length and width compared with those with good contact.^13^ We guided our PFA lesion delivery with ICE and electroanatomic mapping, verifying adequate tissue contact between the Farawave ablation catheter and the PM under ICE. Moreover, given the relatively short time for each PFA application, catheter stability did not represent a significant limitation. We found the Farawave ablation catheter flower configuration useful to ensure catheter contact and stability, as well as to cover a wide range of area while ablating at the PM.

It is important to consider potential collateral damage to the mitral valve apparatus while ablating at the PMs. In the previously mentioned preclinical study in a porcine model, the histologic evaluation at 21 days showed homogeneous fibrotic healing at the PMs with no evidence of mitral valve apparatus damage on ICE.^13^ In addition, other preclinical studies have shown that focal PFA lesions can preserve arterioles and nerve fascicles.^14^ This makes PFA particularly attractive for ablation at the PM site, potentially preserving the mitral valve apparatus structure and function. The risk of coronary vasospasm described when PFA is delivered in close proximity to coronary arteries such as the cavotricuspid isthmus (right coronary artery) would not be expected at the PM location given the relative distance to major epicardial vessels.^15^ Perhaps a considerable risk is catheter entrapment while crossing the mitral valve, although in our case, we mitigated this by using ICE guidance, fluoroscopy, and 3D-electroanatomic mapping. Others have described a retrograde aortic approach for PFA using Farapulse to deliver lesions under the aortic valve and interventricular septum without complications.^16^

There is growing interest in using PFA to ablate arrhythmias originating from the PMs. Using a standard focal radiofrequency ablation catheter connected to a CENTAURI generator (Galvanize Therapeutics, Inc, San Carlos, CA), focal PFA was used to treat PVCs in a recently published case series.^17^ Two patients with posteromedial PM PVCs were included, achieving acute success after ablation in 1 of them (no PVCs in the first 24 hours) but long-term PVC suppression in both of them (reduction of PVC burden, ≥80 %).^17^ This case series described ventricular irritative firing during PFA lesion delivery in approximately half of their patients.^17^ We did not see this phenomenon nor the induction of ventricular arrhythmias during PFA lesion delivery, although we used a different PFA generator and catheter. Initial reports of ablation using the Farapulse PFA for ventricular arrhythmias have shown promising results.^16^^,^^18^, ^19^, ^20^ Recently, Muthu et al^21^ reported a case of anterolateral PM using radiofrequency ablation and a Farapulse PFA system. A total of 6 PFA applications were delivered, with complete PVC suppression following the first PFA lesion delivery and no mitral valve complications.^21^ Our case showed complete PVC suppression after the first PFA application as well, with an additional 9 insurance lesions delivered at the posteromedial PM.

Despite several radiofrequency ablation lesions delivered at the posteromedial PM, we had acute PVC recurrence. This was likely related to the previously mentioned limitations of ablation at this site, and this case highlights the potential use of PFA to successfully ablate arrhythmias at the posteromedial PM and address some of these limitations. It is important to highlight the use of ICE and electroanatomic mapping to guide adequate catheter–tissue contact during our case. Moreover, the relatively short time needed for each lesion delivery, as well as the flower configuration with the Farawave ablation catheter, helped maintain catheter stability at the PM while ablating. We saw both acute and long-term success at PVC suppression using PFA at the posteromedial PM, which was also reflected an improvement in left ventricular systolic function and symptoms. In addition, mitral valve function was preserved after the procedure. Larger studies are needed evaluating the efficacy and safety of available PFA catheters to treat arrhythmias originating from the PMs, and until then caution is advised for routine use of these catheters to ablate in the ventricle. As newer PFA-specific catheters are developed for ablation in the ventricle, it would be interesting to see how other features such as contact force sensing improves ablation outcomes at challenging structures such as the PMs. Finally, studies evaluating postablation cardiac magnetic resonance imaging to assess lesion size and adjacent structures would be valuable as PFA is used to ablate arrhythmias in the ventricle.

## Disclosures

Jonathan Chrispin is a consultant for Biosense Webster and Boston Scientific. He receives educational honorarium from Abbott. Nestor Vasquexz has no disclosures.

## References

[1] Enriquez A., Supple G.E., Marchlinski F.E., Garcia F.C. (2017). How to map and ablate papillary muscle ventricular arrhythmias. Heart Rhythm.

[2] Naksuk N., Kapa S., Asirvatham S.J. (2016). Spectrum of ventricular arrhythmias arising from papillary muscle in the structurally normal heart. Card Electrophysiol Clin.

[3] Batista Napotnik T., Polajzer T., Miklavcic D. (2021). Cell death due to electroporation: a review. Bioelectrochemistry.

[4] Reddy V.Y., Gerstenfeld E.P., Natale A. (2023). Pulsed field or conventional thermal ablation for paroxysmal atrial fibrillation. N Engl J Med.

[5] Verma A., Haines D.E., Boersma L.V. (2023). Pulsed field ablation for the treatment of atrial fibrillation: PULSED AF pivotal trial. Circulation.

[6] Reddy V.Y., Calkins H., Mansour M. (2024). Pulsed field ablation to treat paroxysmal atrial fibrillation: safety and effectiveness in the AdmIRE pivotal trial. Circulation.

[7] Yamada T., Doppalapudi H., McElderry H.T. (2010). Electrocardiographic and electrophysiological characteristics in idiopathic ventricular arrhythmias originating from the papillary muscles in the left ventricle: relevance for catheter ablation. Circ Arrhythm Electrophysiol.

[8] Lin A.N., Shirai Y., Liang J.J. (2020). Strategies for catheter ablation of left ventricular papillary muscle arrhythmias: an institutional experience. JACC Clin Electrophysiol.

[9] Latchamsetty R., Yokokawa M., Morady F. (2015). Multicenter outcomes for catheter ablation of idiopathic premature ventricular complexes. JACC Clin Electrophysiol.

[10] Yamada T., Doppalapudi H., McElderry H.T. (2010). Idiopathic ventricular arrhythmias originating from the papillary muscles in the left ventricle: prevalence, electrocardiographic and electrophysiological characteristics, and results of the radiofrequency catheter ablation. J Cardiovasc Electrophysiol.

[11] Enriquez A., Saenz L.C., Rosso R. (2018). Use of intracardiac echocardiography in interventional cardiology: working with the anatomy rather than fighting it. Circulation.

[12] Rivera S., de la Paz Ricapito M., Espinoza J. (2015). Cryoablation for ventricular arrhythmias arising from the papillary muscles of the left ventricle guided by intracardiac echocardiography and image integration. JACC Clin Electrophysiol.

[13] Nies M., Watanabe K., Kawamura I., Santos-Gallego C.G., Reddy V.Y., Koruth J.S. (2024). Preclinical study of pulsed field ablation of difficult ventricular targets: intracavitary mobile structures, interventricular septum, and left ventricular free wall. Circ Arrhythm Electrophysiol.

[14] Koruth J.S., Kuroki K., Iwasawa J. (2020). Endocardial ventricular pulsed field ablation: a proof-of-concept preclinical evaluation. Europace.

[15] Reddy V.Y., Petru J., Funasako M. (2022). Coronary arterial spasm during pulsed field ablation to treat atrial fibrillation. Circulation.

[16] Martin C.A., Zaw M.T., Jackson N., Morris D., Costanzo P. (2023). First worldwide use of pulsed-field ablation for ventricular tachycardia ablation via a retrograde approach. J Cardiovasc Electrophysiol.

[17] Della Rocca DG., Cespon-Fernandez M., Keelani A. (2024). Focal pulsed field ablation for premature ventricular contractions: a multicenter experience. Circ Arrhythm Electrophysiol.

[18] Katrapati P., Weiss J.P., Baning D., Zawaneh M., Su W., Tung R. (2024). Pulsed field ablation for incessant scar-related ventricular tachycardia: first US report. Heart Rhythm.

[19] Lozano-Granero C., Hirokami J., Franco E. (2023). Case series of ventricular tachycardia ablation with pulsed-field ablation: pushing technology further (into the ventricle). JACC Clin Electrophysiol.

[20] Ouss A., van Stratum L., van der Voort P., Dekker L. (2023). First in human pulsed field ablation to treat scar-related ventricular tachycardia in ischemic heart disease: a case report. J Interv Card Electrophysiol.

[21] Muthu P., Prajapati P., Vemulapalli H., Rodriguez J.F., Raman A., Srivathsan K. (2024). Ablation of premature ventricular complexes originating from papillary muscle using pulsed field energy: the first in USA experience. Heart Rhythm.

