# Supplementary figures and images for: Successful ablation of premature ventricular complexes arising from the posteromedial papillary muscle using Pulse Field Ablation
Source: HeartRhythm Case Rep. 2025 Jan 30;11(4):365–70. doi: 10.1016/j.hrcr.2025.01.013 (PMC12138025; doi:10.1016/j.hrcr.2025.01.013)

## Slide 1
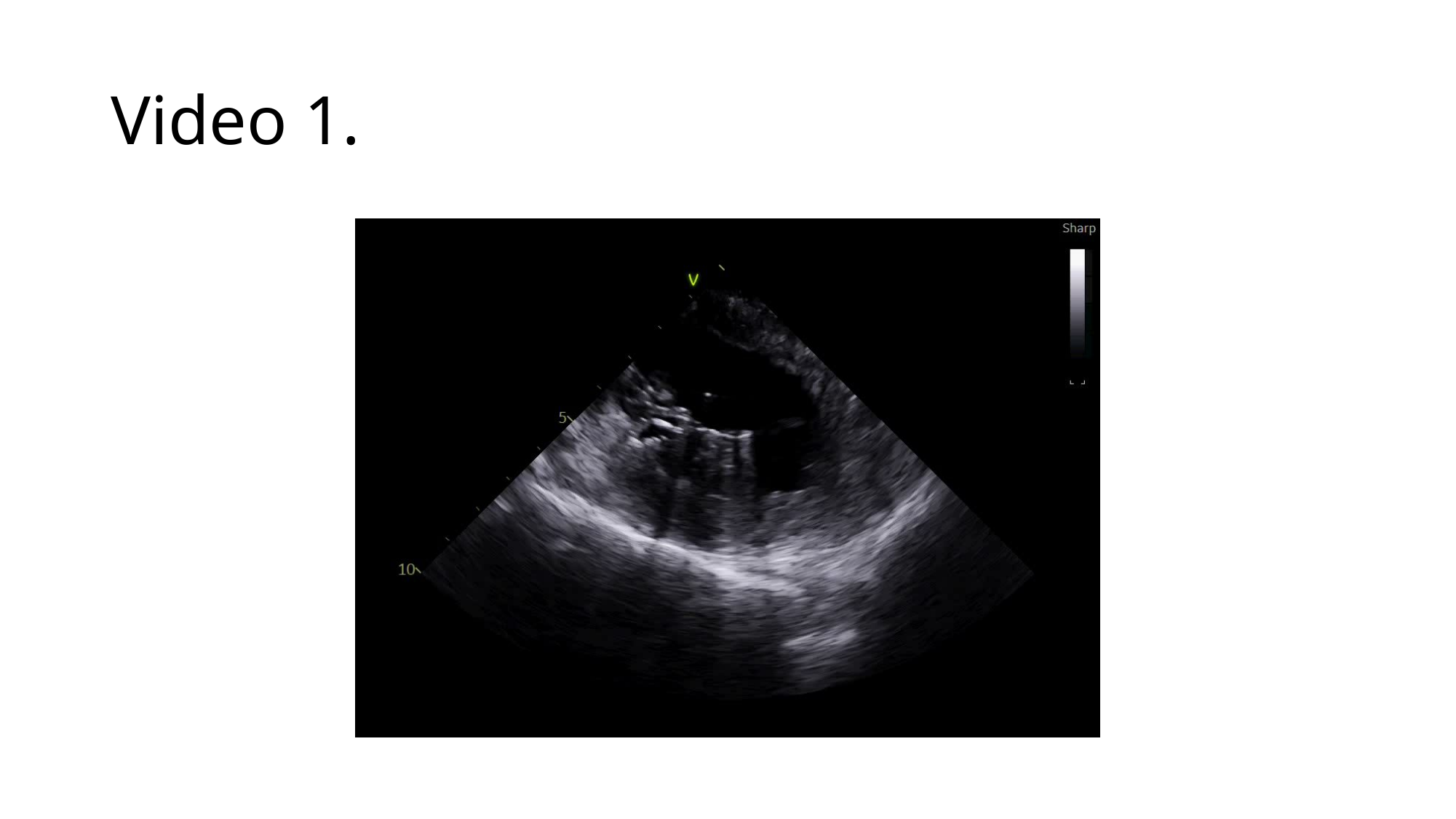

# Video 1.

Supplement: Video 1 [file mmc1.pptx]
